# Long-Term Patient-Reported Outcomes following Oncological Facial Reconstructive Surgery using the FACE-Q Skin Cancer Module

**DOI:** 10.1016/j.jpra.2024.01.003

**Published:** 2024-01-14

**Authors:** J. Nierich, E.M.L. Corten, T. de Jong, M.A.M. Mureau

**Affiliations:** a Department of Plastic & Reconstructive Surgery, Erasmus MC Cancer Institute, University Medical Center Rotterdam, Rotterdam, The Netherlands; b Department of Plastic & Reconstructive Surgery, Radboud University Medical Center, Nijmegen, The Netherlands

**Keywords:** Patient-reported outcomes, quality of life, skin cancer, reconstructive surgery, FACE-Q

## Abstract

**Background:**

Long-term patient-reported outcomes (PROs) of oncological facial reconstructive surgery are unknown.

**Objective:**

The present study aimed to assess long-term PROs and to identify possible correlations between patient and treatment characteristics and long-term PROs.

**Methods & Materials:**

Between 2006 and 2011, 202 patients underwent facial reconstruction after Mohs micrographic surgery for non-melanoma skin cancer at our institution. After 10 years of follow-up, 96 out of the remaining 122 patients completed the FACE-Q Skin Cancer Module.

**Results:**

Patients who were surgically treated for squamous cell carcinoma reported poorer scores on the satisfaction with facial appearance (p=0.038), appraisal of scars (p=0.039) and appearance-related psychosocial distress scales (p=0.036) compared to patients with basal cell carcinoma and lentigo maligna. Finally, female patients reported significantly higher scores on the Cancer Worry Scale than male patients (p=0.047).

**Conclusion:**

Long-term patient satisfaction with respect to their facial appearance and scars after reconstructive surgery for skin cancer was comparable to short-term patient satisfaction, whereas Cancer Worry Scale and psychosocial distress appeared to be slightly higher. Our results can be used to better inform patients on the long-term effects of facial reconstructive surgery on patient satisfaction and quality of life, which are important to improve patient counselling, patient expectation management and shared decision-making.

## Introduction

Skin cancer is a global public health issue. It is the most common malignancy in the Netherlands and despite concerted efforts to reduce skin cancer, its incidence increased from approximately 38,000 new cases in 2007 to approximately 67,000 newly diagnosed patients in 2017.^1^ The majority of these patients were diagnosed with basal or squamous cell carcinoma (BCC and SCC, respectively), which are variants of non-melanoma skin cancer (NMSC). The most important risk factor for NMSC is cumulative sun exposure.^2^^,^^3^ Consequently, most NMSCs occur in the head and neck area, and are located at sites of functional and aesthetic concern, such as the eyelids, nose and lips.^4^ Facial defects that occur following surgical treatment of skin cancer, using Mohs micrographic surgery (MMS), are usually challenging to repair and require a multidisciplinary approach; a plastic and reconstructive surgeon may provide the best care for these patients.^5^^,^^6^ Refinements in plastic surgery techniques have improved functional and aesthetic outcomes following facial reconstructions.^4^^,^^7^^,^^8^

Recently, a paradigm shift has occurred regarding the concept of ‘treatment of disease’, which not only focuses solely on morbidity and mortality, but also includes patient-reported outcomes (PROs) such as health-related quality of life (HRQOL) and satisfaction with outcome. This is crucial to facial skin cancer patients, who require surgery at aesthetic and functional locations and are concerned about negative impacts on their appearance and psychological wellbeing.^9^

Various patient-reported outcome measures (PROMs) have been developed to evaluate the impact of NMSC, such as the Skin Cancer Index (SCI),^10^ Skin Cancer Quality of Life Impact Tool (SCQLIT),^11^ Dermatology Quality of Life Index (DLQI),^12^ and Skindex-29.^13^ However, none of these questionnaires measure patient satisfaction and quality of life after facial reconstructive surgery. The FACE-Q Skin Cancer Module was developed to assess behavioural change, HRQOL, and patient satisfaction in this specific patient population. This validated PROM has already been used in recent studies, which reported short-term PROs up to a maximum of one year in patients who were surgically treated for facial skin cancer.^14^, ^15^, ^16^, ^17^ However, to our knowledge no previous studies have been performed with respect to long-term PROs exceeding one year following oncological facial reconstructive surgery. Tracking long-term PROs is important to improve patient counselling, patient expectation management and shared decision-making. Therefore, the present study aimed to assess long-term PROs after oncological facial reconstructive surgery using the FACE-Q Skin Cancer Module. The second aim was to identify possible correlations between patient and treatment characteristics and long-term PROs.

## Materials and Methods

### Study design and data collection

A single-centre, cross-sectional study design was used. All patients who had undergone facial reconstruction after MMS for NMSC between 2006 and 2011 at the Department of Plastic and Reconstructive surgery of the Erasmus MC Cancer Institute were included. Non-native speakers, patients under 18 years of age, and patients with cognitive impairment, such as dementia, were excluded.

Patient demographics, tumour characteristics and surgical data of our patient cohort had already been collected for a previous study.^6^ These patient data were obtained from the patient electronic medical records and included age, sex, comorbidities, skin cancer specifics (including type, size and pathology), reconstruction details, number of reconstructive operations and postoperative complications.

Patients were selected from the electronic patient files and the GBA (Municipal Personal Records Database) was contacted to enquire if any patients had died. Contact details of the included patients were also obtained from the patient electronic medical records and the informed consent form and FACE-Q questionnaire were sent via postal mail. Patients who did not reply after two weeks were called via telephone and were requested to participate.

### Questionnaire

The Dutch FACE-Q Skin Cancer Module, the Dutch translation of the original version, was used to assess long-term PROs after surgical treatment for facial skin cancer.^18^ The questionnaire was developed using a new psychometric approach defined as the Rasch measurement theory.^19^ It provides a high content validity, measures psychosocial and functional outcomes effectively and has sufficient validity, reliability and responsiveness.^20^ Therefore, clinical changes were captured sufficiently.

The questionnaire was tailored to the patients’ experiences and outcomes following facial skin cancer surgery. Five different domains were measured; appearance-related concerns; psychological functioning (fear of new cancers, recurrence); social functioning (impact on social activities and interaction); adverse problems (pain, swelling) and satisfaction with the experience of care (satisfaction with the treatment team).^9^^,^^20^ The questionnaire focused on the patients’ experiences during the week prior to filling in the questionnaire, thereby preventing systematic errors such as recall bias. The questionnaire takes approximately 15 minutes to complete. The following scales were used in this study: satisfaction with facial appearance, appearance-related psychosocial distress, appraisal of scars, and cancer worry. Each scale can be used independently and consists of 8-10 questions on a Likert-type scale, which are converted into Rasch scores ranging from 0 to 100.^20^ For the domains satisfaction with facial appearance and appraisal of scars, higher scores reflect a better outcome, which is the reverse for the domains of cancer worry and appearance-related psychosocial distress.

### Statistical analysis

Patient data were collected via GemsTracker (Generic Medical Survey Tracker). FACE-Q data were collected via Limesurvey, which is an online survey tool.

De-identified data were analysed. Demographic variables were reported using means or medians with standard deviations (SD) and interquartile ranges (IQR), respectively. Univariate analyses were performed using Pearson's Chi-square tests or Fisher's exact tests for categorical variables. Continuous variables were analysed using Student's *t*-tests or Mann–Whitney U tests. Two-sided p-values <0.05 were considered statistically significant. All data were analysed with IBM SPSS Statistics Version 24.0 (IBM Corp., Armonk, N.Y.).

## Results

Between January 2006 and January 2011, 202 patients underwent oncological facial reconstructive surgery at the Erasmus MC Cancer Institute. In this cohort, 78 patients (38.6%) had died and two patients were being treated for skin cancer. Therefore, the FACE-Q Skin Cancer Module was sent to the remaining 122 patients. Between 15 June 2020 and 8 November 2020, 66 patients completed the questionnaire and returned it by (e)mail, and 30 patients replied by phone, which resulted in a response rate of 78.7%. Nine patients (7.4%) declined study participation and 17 patients (13.9%) did not respond to mail or communication via phone. The median follow-up time between surgery and survey completion was 11 years (IQR, 10-12 years).

Among the patients who completed the survey, 65% were women (n=62) with a mean age of 60 years (SD, 10.5 years) during facial reconstruction. The facial aesthetic units with the most involvement were located centrally, including the mouth, nose and eyelids (Table 1).

**Table 1:** Nonresponse analysis.

Variable	Responders (N=96)	Non-responders (N=26)	p value
Mean age at reconstruction in years (SD)	60 (10.5)	52 (13.8)	***0.005***
Sex			*0.658*
Male	34	8	
Female	62	18	
Tumour size			*0.140*
0-15 mm	32	6	
16-50 mm	28	9	
>50 mm	2	3	
Unknown	34	8	
Affected facial aesthetic unit			*0.143*
Central (nose, eyelids, mouth)	76	17	
Peripheral (cheeks, forehead, chin)	20	9	
Median follow-up in years (IQR)	11 (10-12)	11 (10-12)	*0.865*

A non-response analysis was performed to compare the baseline characteristics between responders and non-responders (Table 1). No statistically significant differences were found between the two groups, except for age during reconstruction with responders being older (p=0.005).

### FACE-Q skin cancer scales

The median scores of the satisfaction with facial appearance scale, appearance-related psychosocial distress scale, appraisal of scars scale, and cancer worry scale in relation to various patient and surgical characteristics are shown in Tables 2 and 3, respectively. Most patient and surgical characteristics, including age at follow-up, recurrence, treatment prior to MMS, facial aesthetic unit and defect size after MMS, did not have a statistically significant relation with the four FACE-Q scale scores. However, women reported higher cancer worry scores and patients who were surgically treated for an SCC reported poorer scores on the satisfaction with facial appearance, appraisal of scars and appearance-related psychosocial distress scales.

**Table 2:** Postoperative Median FACE-Q Skin Cancer Scores (IQR) by Patient Characteristics.

Variable	Satisfaction Facial Appearance	N	p value	Appraisal of Scar	N	p value	Cancer Worry	N	p value	Appearance-related Psychosocial Distress	N	*p* value
*Patient characteristics*												
Age at follow-up			*0.778*			*0.271*			*0.058*			*0.765*
<66 years	70.5 (61.0-100.0)	24		75.0 (65.0-100.0)	24		34.5 (10.5-39.0)	24		19.0 (19.0-33.5)	24	
66-75 years	71.0 (55.0-100.0)	41		91.0 (59.0-100.0)	42		39.0 (21.0-50.0)	42		19.0 (19.0-35.0)	42	
>75 years	76.0 (61.0-100.0)	30		100.0 (71.0-100.0)	30		36.0 (0.0-53.0)	30		19.0 (19.0-32.0)	30	
Sex			*0.194*			0.321			**0.047**			*0.167*
Male	84.5 (61.0-100.0)	34		91.0 (71.0-100.0)	34		23.0 (0.0-39.0)	34		19.0 (19.0-23.0)	34	
Female	67.0 (58.0-100.0)	61		87.5 (59.0-100.0)	62		39.0 (17.0-50.0)	62		19.0 (19.0-35.0)	62	
Tumour			*0.211*			0.223			0.334			*0.365*
Primary	67.0 (53.0-100.0)	43		77.0 (57.5-100.0)	44		39.0 (8.5-50.0)	44		19.0 (19.0-35.0)	44	
Recurrence	80.0 (61.0-100.0)	52		91.0 (65.0-100.0)	52		31.0 (0.0-43.0)	52		19.0 (19.0-28.0)	52	
Treatment prior to MMS			*0.585*			*0.882*			0.632			*0.391*
No	71.0 (53.0-100.0)	38		84.0 (65.0-100.0)	39		39.0 (0.0-48.5)	39		19.0 (19.0-35.0)	39	
Yes	74.0 (61.0-100.0)	57		91.0 (62.0-100.0)	57		33.0 (6.0-44.0)	57		19.0 (19.0-28.0)	57	
Tumour type			***0.038***			**0.039**			*0.731*			***0.036***
BCC	78.0 (61.0-100.0)	85		91.0 (65.0-100.0)	86		33.0 (0.0-47.0)	86		19.0 (19.0-28.0)	86	
SCC	55.0 (53.0-61.0)	5		59.0 (48.0-65.0)	5		39.0 (25.0-55.0)	5		35.0 (35.0-56.0)	5	
Other*	67.0 (67.0-78.0)	5		79.0 (75.0-100.0)	5		36.0 (17.0-42.0)	5		28.0 (19.0-56.0)	5	
Facial aesthetic unit			*0.567*			*0.634*			*0.316*			*0.808*
Central	74.0 (59.5-100.0)	75		87.5 (62.0-100.0)	76		34.5 (0.0-45.5)	76		19.0 (19.0-33.5)	76	
Peripheral	62.5 (59.5-100.0)	20		95.5 (65.0-100.0)	20		36.0 (21.0-48.5)	20		19.0 (19.0-33.5)	20	
Defect size after MMS			*0.304*			*0.408*			*0.949*			*0.531*
Unknown	84.5 (64.0-100.0)	18		100.0 (75.0-100.0)	19		39.0 (21.0-42.0)	19		19.0 (19.0-32.0)	19	
0-15 mm	67.0 (58.0-100.0)	18		91.0 (62.0-100.0)	18		34.5 (0.0-44.0)	18		19.0 (19.0-23.0)	18	
15-50 mm	82.0 (58.0-100.0)	41		91.0 (65.0-100.0)	41		29.0 (0.0-50.0)	41		19.0 (19.0-35.0)	41	
>51 mm	61.0 (50.0-100.0)	18		66.5 (59.0-100.0)	18		27.0 (17.0-44.0)	18		19.0 (19.0-38.0)	18	

IQR: interquartile range. MMS: Mohs micrographic surgery.

⁎ Fibrous fibroadenoma, Merkel cell carcinoma, lentigo maligna and eccrine nevus.

**Table 3:** Postoperative Median FACE-Q Skin Cancer Scores (IQR) by Surgical Characteristics.

Variable	Satisfaction Facial Appearance	N	p value	Appraisal of Scar	N	p value	Cancer Worry	N	p value	Appearance-related Psychosocial Distress	N	p value
*Surgery characteristics*												
Type of reconstruction			*0.535*			*0.182*			*0.300*			*0.447*
Primary closure	100.0 (77.0-100.0)	7		100.0 (74.5-100.0)	7		29.0 (10.5-48.5)	7		19.0 (19.0-19.0)	7	
Skin graft	64.0 (61.0-93.5)	11		95.5 (79.5-100.0)	12		23.0 (8.5-37.5)	12		19.0 (19.0-25.5)	12	
Local flap	76.0 (57.0-100.0)	28		69.5 (59.0-100.0)	28		39.0 (23.0-53.0)	28		19.0 (19.0-40.0)	28	
Regional flap	67.0 (55.0-100.0)	49		91.0 (65.0-100.0)	49		33.0 (0.0-44.0)	49		19.0 (19.0-35.0)	49	
Reoperations			*0.799*			*0.545*			*0.742*			*0.471*
None	74.0 (61.0-100.0)	49		87.5 (65.0-100.0)	50		33.0 (17.0-47.0)	*50*		19.0 (19.0-35.0)	50	
One or more	72.5 (55.0-100.0)	46		91.0 (62.0-100.0)	46		36.0 (0.0-44.0)	*46*		19.0 (19.0-32.0)	46	
Complication			*0.136*			*0.874*			*0.617*			*0.152*
No	74.0 (61.0-100.0)	80		91.0 (65.0-100.0)	81		33.0 (6.0-44.0)	81		19.0 (19.0-32.0)	81	
Yes	61.0 (55.0-84.5)	15		100.0 (54.5-100.0)	15		39.0 (0.0-54.0)	15		23.0 (19.0-49.0)	15	
Follow-up			*0.400*			*0.640*			*0.634*			*0.150*
≤ 11 years	69.0 (58.0-100.0)	58		84.0 (62.0-100.0)	59		36.0 (03.0-50.0)	59		19.0 (19.0-30.0)	59	
> 11 years	82.0 (61.0-100.0)	37		91.0 (65.0-100.0)	37		33.0 (0.0-44.0)	37		19.0 (19.0-35.0)	37	

## Discussion

To our knowledge, this is the first study to investigate long-term satisfaction with facial appearance and scars more than one year following oncological facial reconstructive surgery, using the FACE-Q Skin Cancer Module. Even though no formal comparison between short- and long-term data was performed, the results of this study appeared to be comparable to patient satisfaction reported after one to two years and could be used for counselling patients who need facial reconstructive surgery after MMS.^14^, ^15^, ^16^, ^17^^,^^21^

Previously reported short-term satisfaction with facial appearance and satisfaction with scars scores ranged from 60 to 81 and from 65 to 82, respectively.^14^, ^15^, ^16^, ^17^^,^^21^ In addition, the mean short-term cancer worry score was approximately 25 and psychosocial distress ranged from 17 to 39. However, cancer worry and psychosocial distress scores appeared to be slightly higher for long-term compared to the scores for short-term satisfaction.^14^, ^15^, ^16^, ^17^^,^^21^, ^22^ Now, we know that the long-term changes in PROs are relatively small. Therefore, future studies can focus on the first two years after reconstruction, saving valuable time and money associated with long-term follow-up.

In addition, patients treated for SCCs reported less satisfaction with facial appearance and scars, which may possibly lead to increased distress. In our study, 40% of the patients with SCC were treated with a skin graft compared to 17% of the patients with BCC. Facial reconstruction with local flaps generally leads to higher patient satisfaction than the use of skin grafts owing to their poor colour and texture match next to the surrounding normal skin, and this could explain the less favourable PROs found in patients with SCC.^23^ However, no significant differences were found in PROs between different types of reconstruction, which may be due to the relatively small sample size of the primary closure and skin grafts groups.

Furthermore, in this study we found that female patients had higher cancer worry scores than male patients, which is in line with the results of a previous study.^24^ Gender differences may influence both the process and motivation for information retrieval, consultation with other people, and impact on intentions and behaviour.^24^ Information seeking seemed to affect the amount of worry and men were generally less likely to seek cancer information compared to women.^24^

Other studies found that anatomic location of the tumour, type of reconstruction and patient characteristics may have an impact on patient satisfaction. Moreover, female sex and a younger age were independent predictors for lower postoperative facial aesthetic satisfaction.^16^^,^^21^ Women who experienced greater difficulty in adapting to facial cancer, placed a higher value on facial aesthetics, and had worse appearance-related quality of life at baseline.^16^^,^^21^ However, these predictors were not found in our study, which might predominantly be related to the longer follow-up time between surgery and survey completion. Previous FACE-Q studies showed short-term outcomes with a follow-up time varying between 16 days until 59 weeks after surgery.^14^, ^15^, ^16^, ^17^^,^^21^ In 2019, Vaidya et al. showed that psychosocial distress was higher until three months after surgery, after which it significantly improved over time.

We could not draw conclusions regarding the influence of younger age as a predictor for poorer outcomes, probably due to the relatively older age of our responding group. Older age is associated with a more favourable cosmetic outcome, because the presence of wrinkles, skin folds, and irregular contours in older patients conceal surgical scars better.^15^^,^^16^^,^^21^ In addition, greater skin laxity seen in older patients may provide additional local skin for repair, ultimately leading to less tension on the wound and more often primary closure without the need for skin grafts or local flaps.

### Limitations

Limitations of this study include the possibility of referral bias since this study was conducted in a tertiary care centre. Enrolment of patients with complex tumours and reconstructions makes our results less generalisable to all patients with skin cancer. Moreover, including patients via email, postal mail or telephone could have led to interview bias. However, prior research showed that the use of multiple communication techniques such as email, postal mail or telephone increases the responses to research questionnaires.^25^^,^^26^

In addition, due to the relatively small sample size, conclusions have to be made with caution, as they may overestimate or underestimate the current associations found.^27^

Finally, selection bias occurred in terms of the age of patients, with the group of responders having a higher age at reconstruction. As older patients are less affected by changes in their facial appearance, which leads to higher scores on patient satisfaction, the outcomes based on the results of the present study might be an overestimation.^16^ To definitively assess long-term patient-reported outcomes, a large prospective study should ideally be undertaken to include patients with skin cancer of various ages, particularly those with large facial reconstructions.

## Conclusions

Long-term patient satisfaction with regard to facial appearance and scars after reconstructive surgery for facial skin cancer treatment appears to be comparable to short-term patient satisfaction, whereas cancer worry and psychosocial distress seem to be slightly higher. In addition, patients with SCC tend to have less satisfaction with facial appearance and scars and more psychosocial distress. Finally, female patients reported higher cancer worry scores than male patients. Using our results, it is now possible to better inform patients on the long-term effects of facial reconstructive surgery in terms of satisfaction and quality of life, which is important to improve patient counselling, patient expectation management and shared decision-making. For example, intensive support should be offered to young female patients with facial reconstruction since they tend to report higher psychological distress and cancer worry on the long-term.

## Declaration of interest statement

The authors report there are no competing interests to declare.
